# *catena*-Poly[[aqua­bis­(pyridine-κ*N*)copper(II)]-μ-2,2′-(*p*-phenyl­enedi­oxy)diacetato-κ^2^ *O*:*O*′]

**DOI:** 10.1107/S1600536810036469

**Published:** 2010-09-18

**Authors:** Xiu-Mei Zhang, Ya-Feng Li

**Affiliations:** a Department of X-ray, First Hospital, Jilin University, Changchun 130021, People’s Republic of China; b School of Chemical Engineering, Changchun University of Technology, Changchun 130012, People’s Republic of China

## Abstract

In the title compound, [Cu(C_10_H_8_O_6_)(C_5_H_5_N)_2_(H_2_O)]_*n*_, the Cu atom is five-coordinated by two O atoms from two carboxyl­ate groups of two different 2,2′-(*p*-phenyl­enedi­oxy)diacetate ligands, two N atoms from two pyridine mol­ecules and one water O atom. The geometry is square-pyramidal with the water O atom occupying the apical position. The carboxyl­ate group bridges adjacent Cu atoms, forming an infinite zigzag chain extending parallel to [001]. The chains are linked into layers by O—H⋯O hydrogen bonds. The Cu and water O atoms lie on special positions of site symmetry 2.

## Related literature

For the isotypic zinc analog, see: Hong *et al.* (2005 ▶).
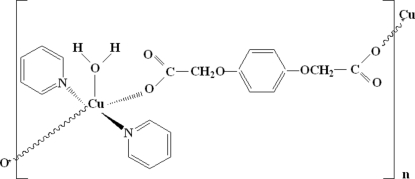


## Experimental

### 

#### Crystal data

[Cu(C_10_H_8_O_6_)(C_5_H_5_N)_2_(H_2_O)]
*M* *_r_* = 463.93
Monoclinic, 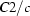

*a* = 15.363 (4) Å
*b* = 6.0888 (12) Å
*c* = 21.896 (6) Å
β = 103.67 (3)°
*V* = 1990.2 (8) Å^3^
*Z* = 4
Mo *K*α radiation
μ = 1.14 mm^−1^
*T* = 298 K
0.12 × 0.11 × 0.09 mm

#### Data collection

Rigaku R-AXIS RAPID diffractometer
Absorption correction: multi-scan (*ABSCOR*; Higashi, 1995 ▶) *T*_min_ = 0.875, *T*_max_ = 0.907
7227 measured reflections
1737 independent reflections
1096 reflections with *I* > 2σ(*I*)
*R*_int_ = 0.119

#### Refinement

*R*[*F* ^2^ > 2σ(*F* ^2^)] = 0.071
*wR*(*F* ^2^) = 0.123
*S* = 1.08
1737 reflections
140 parameters
H atoms treated by a mixture of independent and constrained refinement
Δρ_max_ = 0.32 e Å^−3^
Δρ_min_ = −0.38 e Å^−3^

### 

Data collection: *PROCESS-AUTO* (Rigaku, 1998 ▶); cell refinement: *PROCESS-AUTO*; data reduction: *CrystalStructure* (Rigaku/MSC, 2002 ▶); program(s) used to solve structure: *SHELXS97* (Sheldrick, 2008 ▶); program(s) used to refine structure: *SHELXL97* (Sheldrick, 2008 ▶); molecular graphics: *DIAMOND* (Brandenburg, 2000 ▶); software used to prepare material for publication: *SHELXL97*.

## Supplementary Material

Crystal structure: contains datablocks I, global. DOI: 10.1107/S1600536810036469/ng5029sup1.cif

Structure factors: contains datablocks I. DOI: 10.1107/S1600536810036469/ng5029Isup2.hkl

Additional supplementary materials:  crystallographic information; 3D view; checkCIF report

## Figures and Tables

**Table 1:** Hydrogen-bond geometry (Å, °)

*D*—H⋯*A*	*D*—H	H⋯*A*	*D*⋯*A*	*D*—H⋯*A*
O1*W*—H1*A*⋯O2^i^	0.81 (6)	1.87 (6)	2.677 (5)	174 (7)

Symmetry code: (i) 
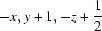
.

**Fig. 1.**
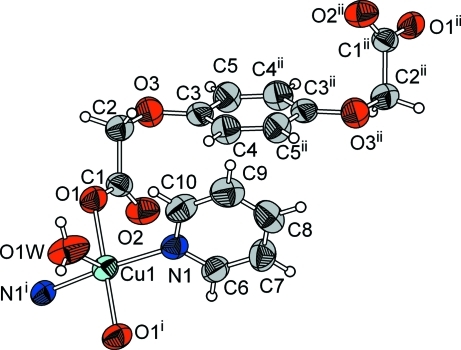
The unit cell of (I), showing the atomic labelling scheme and displacement ellipsoids at the 50% probability level. [Symmetry codes: (i) -x, y, 0.5 - z; (ii) -x, 1 - y, -z.]

**Fig. 2.**
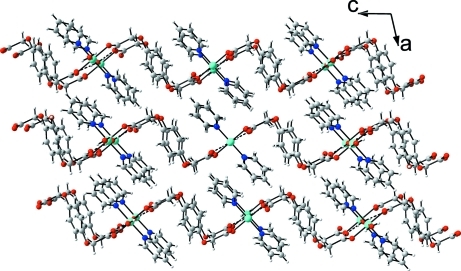
The ball-stick plot of (I), displaying the zigzag chain along (001) direction composed of briging the Cu cation with monodentate µ2-BDOA. Cu is shown in the cyan, O in red, N in blue and C in grey.

## supplementary crystallographic information

### Comment

The metal-organic framework, which is formed by carboxylate ligand as the strut and transition metal as the node, has recently attracted more attentions owing to the potential applications of catalyst and H-storage. In this paper, flexible ligand-BDOA as the strut bridges the Cu cations to result in the formation of infinite zigzag chain of [Cu(py)_2_(H_2_O)BDOA]_n_.

The asymmetrical unit of [Cu(py)_2_(H_2_O)BDOA]_n_ (py=pyridine, BDOA=benzene-1,4-dioxyacetate) which is isostructural with of [Zn(py)_2_(H_2_O)BDOA]_n_ (Hong, *et al.*, 2005), is composed of one half of Cu cation, one half of BDOA, one half of water molecule and one pyridine molecule (Fig.1). Five-coordinated Cu cation lies in the basal position of pyramid constructed by two O atoms from two carboxyl groups of two different BDOA, two nitrogen atoms of two pyridines and one water oxygen atom situated at the apical position. The bond distances of Cu—N, Cu—O and Cu—Ow are 2.109 (23) Å, 1.965 (35) Å and 2.192 (6) Å, respectively. The bond angles of O—Cu—O and N—Cu—O are 179.85 (14)° and 167.68 (18)°, respectively. Cu and water oxygen lie at 2-fold axis and BDOA at the inversion center.

The monodentate µ_2_-BDOA bridges the adjacent Cu cations to form the infinite zigzag chain along (001) direction. The H-bonds of Ow—H···O (free oxygen of carboxyl) link the adjacent chains to two-dimensional layer (*bc* planar), which is packed by the ver dan Waals force (Fig.2).

### Experimental

(I) was synthesized under hydrothermal condition. In a typically route, H_2_BDOA (0.22 g, 1 mmol) was dissolved in 10 ml deionized water under stirring, and then pyridine (1.6 ml, 20 mmol) and Cu(Ac)_2_^.^3H_2_O (0.235 g, 1 mmol) were added to a blue solution. After continuously stirred for 1 h, the solution with the molar ratio of H_2_BDOA: 20py: Cu(Ac)_2_^.^3H_2_O: 555H_2_O was transferred into 23 ml autoclave and heated at 438 K for 5 days. After naturally cooling to room temperature, blue block product was collected by filtration as a single phase.

### Refinement

Water H atoms were located in a difference Fourier map and were refined with O—H = 0.82 (2) Å, H···H = 1.37 (2) Å and *U*_iso_(H) = 1.2Ueq(O). The remaining H-atoms were placed in calculated positions (C—H (phenyl and pyridine ring) = 0.93 Å, C—H (methylene) = 0.97 Å) and were included in the refinement in the riding-model approximation, with U(H) = 1.2Ueq(C).

### Crystal data

**Table:**

[Cu(C_10_H_8_O_6_)(C_5_H_5_N)_2_(H_2_O)]	*F*(000) = 956
*M**_r_* = 463.93	*D*_x_ = 1.548 Mg m^−^^3^
Monoclinic, *C*2/*c*	Mo *K*α radiation, λ = 0.71073 Å
Hall symbol: -C 2yc	Cell parameters from 2000 reflections
*a* = 15.363 (4) Å	θ = 3.6–25°
*b* = 6.0888 (12) Å	µ = 1.14 mm^−^^1^
*c* = 21.896 (6) Å	*T* = 298 K
β = 103.67 (3)°	Block, blue
*V* = 1990.2 (8) Å^3^	0.12 × 0.11 × 0.09 mm
*Z* = 4	

### Data collection

**Table:**

Rigaku R-AXIS RAPID diffractometer	1737 independent reflections
Radiation source: fine-focus sealed tube	1096 reflections with *I* > 2σ(*I*)
graphite	*R*_int_ = 0.119
Detector resolution: 10.00 pixels mm^-1^	θ_max_ = 25.0°, θ_min_ = 3.6°
ω scans	*h* = −18→18
Absorption correction: multi-scan (*ABSCOR*; Higashi, 1995)	*k* = −7→6
*T*_min_ = 0.875, *T*_max_ = 0.907	*l* = −25→25
7227 measured reflections	

### Refinement

**Table:**

Refinement on *F*^2^	Primary atom site location: structure-invariant direct methods
Least-squares matrix: full	Secondary atom site location: difference Fourier map
*R*[*F*^2^ > 2σ(*F*^2^)] = 0.071	Hydrogen site location: inferred from neighbouring sites
*wR*(*F*^2^) = 0.123	H atoms treated by a mixture of independent and constrained refinement
*S* = 1.08	*w* = 1/[σ^2^(*F*_o_^2^) + (0.0254*P*)^2^ + 4.0919*P*] where *P* = (*F*_o_^2^ + 2*F*_c_^2^)/3
1737 reflections	(Δ/σ)_max_ < 0.001
140 parameters	Δρ_max_ = 0.32 e Å^−^^3^
0 restraints	Δρ_min_ = −0.38 e Å^−^^3^

### Special details

**Table:**

Geometry. All e.s.d.'s (except the e.s.d. in the dihedral angle between two l.s. planes) are estimated using the full covariance matrix. The cell e.s.d.'s are taken into account individually in the estimation of e.s.d.'s in distances, angles and torsion angles; correlations between e.s.d.'s in cell parameters are only used when they are defined by crystal symmetry. An approximate (isotropic) treatment of cell e.s.d.'s is used for estimating e.s.d.'s involving l.s. planes.
Refinement. Refinement of *F*^2^ against ALL reflections. The weighted *R*-factor *wR* and goodness of fit *S* are based on *F*^2^, conventional *R*-factors *R* are based on *F*, with *F* set to zero for negative *F*^2^. The threshold expression of *F*^2^ > σ(*F*^2^) is used only for calculating *R*-factors(gt) *etc*. and is not relevant to the choice of reflections for refinement. *R*-factors based on *F*^2^ are statistically about twice as large as those based on *F*, and *R*- factors based on ALL data will be even larger.

### Fractional atomic coordinates and isotropic or equivalent isotropic displacement parameters (Å^2^)

**Table:**

	*x*	*y*	*z*	*U*_iso_*/*U*_eq_	
Cu1	0.0000	1.02668 (15)	0.2500	0.0480 (4)	
O1	−0.0864 (2)	1.0271 (7)	0.16798 (16)	0.0584 (10)	
O2	−0.0691 (3)	0.6662 (7)	0.15674 (17)	0.0679 (12)	
O3	−0.1479 (3)	0.6794 (7)	0.03254 (17)	0.0656 (12)	
C1	−0.0948 (4)	0.8473 (11)	0.1365 (2)	0.0506 (15)	
C2	−0.1406 (4)	0.8781 (10)	0.0675 (2)	0.0581 (16)	
H2A	−0.2001	0.9377	0.0643	0.070*	
H2B	−0.1070	0.9840	0.0493	0.070*	
C3	−0.0715 (4)	0.5975 (10)	0.0191 (2)	0.0528 (15)	
C4	0.0125 (4)	0.6894 (10)	0.0344 (3)	0.0618 (17)	
H4	0.0218	0.8182	0.0579	0.074*	
C5	−0.0824 (4)	0.4048 (10)	−0.0158 (3)	0.0605 (17)	
H5	−0.1385	0.3387	−0.0266	0.073*	
N1	0.0988 (3)	0.9911 (8)	0.20420 (18)	0.0503 (12)	
C6	0.1565 (4)	0.8241 (10)	0.2156 (3)	0.0571 (16)	
H6	0.1512	0.7228	0.2463	0.068*	
C7	0.2226 (4)	0.7939 (11)	0.1847 (3)	0.0681 (18)	
H7	0.2605	0.6731	0.1937	0.082*	
C8	0.2327 (4)	0.9417 (13)	0.1405 (3)	0.0713 (19)	
H8	0.2787	0.9270	0.1198	0.086*	
C9	0.1732 (5)	1.1140 (12)	0.1271 (3)	0.075 (2)	
H9	0.1771	1.2156	0.0962	0.090*	
C10	0.1086 (4)	1.1333 (10)	0.1598 (3)	0.0659 (18)	
H10	0.0693	1.2514	0.1508	0.079*	
O1W	0.0000	1.3867 (10)	0.2500	0.085 (2)	
H1A	0.023 (5)	1.463 (10)	0.280 (3)	0.102*	

### Atomic displacement parameters (Å^2^)

**Table:**

	*U*^11^	*U*^22^	*U*^33^	*U*^12^	*U*^13^	*U*^23^
Cu1	0.0449 (6)	0.0454 (6)	0.0508 (6)	0.000	0.0055 (4)	0.000
O1	0.047 (2)	0.067 (3)	0.055 (2)	0.003 (2)	0.0017 (18)	−0.007 (2)
O2	0.082 (3)	0.057 (3)	0.059 (3)	0.000 (2)	0.006 (2)	0.007 (2)
O3	0.052 (3)	0.080 (3)	0.062 (2)	−0.005 (2)	0.009 (2)	−0.016 (2)
C1	0.040 (4)	0.069 (5)	0.042 (3)	−0.005 (3)	0.008 (3)	0.001 (3)
C2	0.055 (4)	0.064 (4)	0.052 (3)	0.004 (3)	0.005 (3)	−0.001 (3)
C3	0.043 (4)	0.070 (4)	0.042 (3)	−0.001 (3)	0.004 (3)	−0.002 (3)
C4	0.057 (5)	0.065 (4)	0.060 (4)	−0.010 (3)	0.008 (3)	−0.015 (3)
C5	0.042 (4)	0.072 (4)	0.067 (4)	−0.012 (3)	0.013 (3)	−0.006 (3)
N1	0.042 (3)	0.057 (3)	0.048 (3)	−0.005 (3)	0.004 (2)	0.001 (2)
C6	0.051 (4)	0.062 (4)	0.057 (4)	0.005 (3)	0.010 (3)	0.011 (3)
C7	0.054 (5)	0.073 (5)	0.081 (5)	0.005 (4)	0.024 (4)	0.006 (4)
C8	0.049 (4)	0.103 (6)	0.066 (4)	−0.026 (4)	0.020 (3)	−0.020 (4)
C9	0.063 (5)	0.093 (5)	0.070 (4)	−0.018 (4)	0.018 (4)	0.017 (4)
C10	0.053 (4)	0.069 (4)	0.070 (4)	−0.004 (3)	0.004 (3)	0.015 (4)
O1W	0.114 (6)	0.041 (4)	0.078 (5)	0.000	−0.021 (4)	0.000

### Geometric parameters (Å, °)

**Table:**

Cu1—O1	1.964 (3)	C4—H4	0.9300
Cu1—O1^i^	1.964 (3)	C5—C4^ii^	1.362 (8)
Cu1—N1	2.019 (4)	C5—H5	0.9300
Cu1—N1^i^	2.019 (4)	N1—C6	1.333 (7)
Cu1—O1W	2.192 (6)	N1—C10	1.338 (7)
O1—C1	1.284 (6)	C6—C7	1.359 (8)
O2—C1	1.219 (6)	C6—H6	0.9300
O3—C3	1.370 (6)	C7—C8	1.357 (8)
O3—C2	1.422 (6)	C7—H7	0.9300
C1—C2	1.520 (7)	C8—C9	1.377 (9)
C2—H2A	0.9700	C8—H8	0.9300
C2—H2B	0.9700	C9—C10	1.357 (8)
C3—C4	1.373 (7)	C9—H9	0.9300
C3—C5	1.388 (8)	C10—H10	0.9300
C4—C5^ii^	1.362 (8)	O1W—H1A	0.81 (6)
			
O1—Cu1—O1^i^	179.8 (3)	C5^ii^—C4—H4	119.4
O1—Cu1—N1	88.32 (15)	C3—C4—H4	119.4
O1^i^—Cu1—N1	91.70 (15)	C4^ii^—C5—C3	121.3 (6)
O1—Cu1—N1^i^	91.70 (15)	C4^ii^—C5—H5	119.4
O1^i^—Cu1—N1^i^	88.32 (15)	C3—C5—H5	119.4
N1—Cu1—N1^i^	167.7 (3)	C6—N1—C10	116.5 (5)
O1—Cu1—O1W	89.92 (13)	C6—N1—Cu1	122.2 (4)
O1^i^—Cu1—O1W	89.92 (13)	C10—N1—Cu1	121.3 (4)
N1—Cu1—O1W	96.16 (14)	N1—C6—C7	123.5 (6)
N1^i^—Cu1—O1W	96.16 (14)	N1—C6—H6	118.3
C1—O1—Cu1	116.8 (4)	C7—C6—H6	118.3
C3—O3—C2	117.5 (5)	C8—C7—C6	119.4 (6)
O2—C1—O1	126.4 (5)	C8—C7—H7	120.3
O2—C1—C2	120.4 (5)	C6—C7—H7	120.3
O1—C1—C2	113.1 (5)	C7—C8—C9	118.3 (6)
O3—C2—C1	113.0 (5)	C7—C8—H8	120.8
O3—C2—H2A	109.0	C9—C8—H8	120.8
C1—C2—H2A	109.0	C10—C9—C8	119.0 (6)
O3—C2—H2B	109.0	C10—C9—H9	120.5
C1—C2—H2B	109.0	C8—C9—H9	120.5
H2A—C2—H2B	107.8	N1—C10—C9	123.3 (6)
O3—C3—C4	127.1 (6)	N1—C10—H10	118.4
O3—C3—C5	115.3 (5)	C9—C10—H10	118.4
C4—C3—C5	117.6 (6)	Cu1—O1W—H1A	125 (5)
C5^ii^—C4—C3	121.1 (6)		
			
N1—Cu1—O1—C1	66.8 (4)	O1^i^—Cu1—N1—C6	56.9 (4)
N1^i^—Cu1—O1—C1	−100.9 (4)	N1^i^—Cu1—N1—C6	−33.0 (4)
O1W—Cu1—O1—C1	162.9 (4)	O1W—Cu1—N1—C6	147.0 (4)
Cu1—O1—C1—O2	15.7 (8)	O1—Cu1—N1—C10	55.6 (4)
Cu1—O1—C1—C2	−163.2 (3)	O1^i^—Cu1—N1—C10	−124.3 (4)
C3—O3—C2—C1	−73.6 (6)	N1^i^—Cu1—N1—C10	145.9 (4)
O2—C1—C2—O3	−0.3 (8)	O1W—Cu1—N1—C10	−34.1 (4)
O1—C1—C2—O3	178.6 (5)	C10—N1—C6—C7	−0.1 (8)
C2—O3—C3—C4	−1.8 (8)	Cu1—N1—C6—C7	178.8 (4)
C2—O3—C3—C5	−179.3 (4)	N1—C6—C7—C8	1.3 (10)
O3—C3—C4—C5^ii^	−177.2 (5)	C6—C7—C8—C9	−2.2 (9)
C5—C3—C4—C5^ii^	0.3 (9)	C7—C8—C9—C10	2.0 (9)
O3—C3—C5—C4^ii^	177.5 (5)	C6—N1—C10—C9	−0.1 (8)
C4—C3—C5—C4^ii^	−0.3 (9)	Cu1—N1—C10—C9	−179.0 (5)
O1—Cu1—N1—C6	−123.2 (4)	C8—C9—C10—N1	−0.9 (10)

Symmetry codes: (i) −*x*, *y*, −*z*+1/2; (ii) −*x*, −*y*+1, −*z*.

### Hydrogen-bond geometry (Å, °)

**Table:**

*D*—H···*A*	*D*—H	H···*A*	*D*···*A*	*D*—H···*A*
O1W—H1A···O2^iii^	0.81 (6)	1.87 (6)	2.677 (5)	174 (7)

Symmetry codes: (iii) −*x*, *y*+1, −*z*+1/2.
